# Miscellany

**Published:** 1891-03

**Authors:** 


### Miscellany.

Vick's Floral Guide for 1891.—No lover of a fine plant or garden can afford to be without a copy. It is an elegant book of over 100 pages, $8\frac{1}{4}$ x $10\frac{1}{2}$ inches, beautiful colored illustrations of sunrise amaranthus, hydrangea and potatoes. Instructions for planting, cultivating, etc. Full list of everything that can be desired in the way of vegetable and flower seeds, plants, bulbs, etc. Also full particulars regarding the cash prizes of $1,000 and $200. The novelties have been tested and found worthy of cultivation. We hope it will be our good luck to see the Nellie Lewis carnation and taste the Grand Rapids lettuce. It costs nothing, because the 10 cents you send for it can be deducted from the first order forwarded. We advise our friends to secure a copy of James Vick, Seedsman, Rochester, N. Y.

Recent Medical Patents. — Liquid-evaporating apparatus (3 patents), J. A. Morrell, Lansdale, Pa. ; Evaporating liquids, J. A. Morrell, Lansdale, Pa. ; Insecticide, F. Jones, Morse, Kan. ; Obstetrical forceps, A. B. Lyman, Baltimore, Md. ; Making vegetable pepsine, V. Marcano, Caracas, Venezuela ; Making peptonized meat, V. Marcano, Caracas, Venezuela; Making peptones, V. Marceno, Caracas, Venezuela ; Concentrating solutions (2 patents), J. A. Morrell, Lansdale, Pa. ; Syringe (2 patents), W. Guptill, Boston, Mass. ; Artificial tooth, F. T. Van Woert, Brooklyn, N. Y. ; Dose-measuring bottle, H. S. Herrick, San Francisco, Cal. ; Dental-pluggers, J. L. Mewborn, Memphis, Tenn. ; Disinfecting apparatus, F. J. Mitchell, New York, N. Y. ; Nursing-bottle holder, J. Von Huppman-Valbella, New York, N. Y. ; Forceps for dental-wedges, G. J. Davison, Richmond, Va. ; Surgical bandage winder, W. Gilfillan, Steinway, N. Y. ; Vaginal syringe, J. R. Trott, Morrisonville, Ill. ; Ammonia-still, F. Kaiser, Knoxville, Tenn. ; Capsule-press, F. W. Perry, Philadelphia, Pa. ; Liniment, D. Meinen, Warrenton, Tex. ; Syringe, E. Bartsch, San Francisco, Cal. ; Device for facilitating the taking of pills, J. Yates, London, England.

Chas. J. Gooch, *Patent Attorney, Washington, D. C.*

---

THE AMERICAN ASSOCIATION OF OBSTETRICIANS AND GYNECOLOGISTS.

Office of the Secretary,
284 Franklin Street, Buffalo, N. Y.
February 20, 1891.

The announcement is made to the profession that the Annual Transactions, Vol. III., 1890, of this Association is now ready for delivery. It is a handsome book, standard octavo in size, containing over 400 pages, and is profusely illustrated with plates, photo-engravings and wood-cuts. The edition is limited, and orders must be placed at once. Price, cloth, $5 ; half Russia, $6.

William Warren Potter, M. D., *Secretary.*

284 Franklin Street, Buffalo, N. Y.

